# Aquatic Insects of New York Salt Marsh Associated with Mosquito Larval Habitat and their Potential Utility as Bioindicators

**DOI:** 10.1673/031.011.17201

**Published:** 2011-12-19

**Authors:** Ilia Rochlin, Mary E. Dempsey, Tom Iwanejko, Dominick V. Ninivaggi

**Affiliations:** ^1^ Division of Vector Control, Suffolk County Department of Public Works, 335 Yaphank Avenue, Yaphank, NY 11980–9744, USA; ^2^ Suffolk County Department of Environment and Energy, 335 Yaphank Avenue, Yaphank, NY 11980–9744, USA

**Keywords:** aquatic community, biological assessment, Bti, environmental impact, food web methoprene

## Abstract

The aquatic insect fauna of salt marshes is poorly characterized, with the possible exception of biting Diptera. Aquatic insects play a vital role in salt marsh ecology, and have great potential importance as biological indicators for assessing marsh health. In addition, they may be impacted by measures to control mosquitoes such as changes to the marsh habitat, altered hydrology, or the application of pesticides. Given these concerns, the goals of this study were to conduct the first taxonomic survey of salt marsh aquatic insects on Long Island, New York, USA and to evaluate their utility for non-target pesticide impacts and environmental biomonitoring. A total of 18 species from 11 families and five orders were collected repeatedly during the five month study period. Diptera was the most diverse order with nine species from four families, followed by Coleoptera with four species from two families, Heteroptera with three species from three families, then Odonata and the hexapod Collembola with one species each. Water boatmen, *Trichocorixa verticalis* Fieber (Heteroptera: Corixidae) and a shore fly, *Ephydra subopaca* Loew (Diptera: Ephydridae), were the two most commonly encountered species. An additional six species; *Anurida maritima* Guérin-Méneville (Collembola: Neanuridae), *Mesovelia mulsanti* White (Heteroptera: Mesovelidae), *Enochrus hamiltoni* Horn (Coleoptera: Hydrophilidae), *Tropisternus quadristriatus* Horn (Coleoptera: Hydrophilidae), *Dasyhelea pseudocincta* Waugh and Wirth (Diptera: Ceratopogonidae), and *Brachydeutera argentata* Walker (Diptera: Ephydridae), were found regularly. Together with the less common *Erythrodiplax berenice* Drury (Odonata: Libellulidae), these nine species were identified as the most suitable candidates for pesticide and environmental impact monitoring due to abundance, position in the food chain, and extended seasonal occurrence. This study represents a first step towards developing an insectbased index of biological integrity for salt marsh health assessment.

## Introduction

About 300 species of insects have been described from salt marshes worldwide, but little is known about their basic biology and ecology due to their perceived lack of importance. Biting Diptera species are one notable exception (Foster and Treherne 1976; Ward 1992). Horse and deer flies, biting midges, and especially mosquitoes are abundant in the salt marsh environment and present a significant public health problem in many parts of the world, thus attracting attention and resources for their study and control. While not important for public health, other salt marsh insects may play a vital role in the marsh ecology and food web (Teal and Teal 1969). Aquatic insects inhabiting salt marshes may also be of a considerable practical interest as potential bioindicators for evaluating the wetland health (EPA 2002a). Surveys of salt marsh aquatic insects have been conducted in many northeastern and mid Atlantic states (Wall 1973; Campbell and Denno 1978; Kelts 1979; Robert and Matta 1984; MacKenzie 2005), but not in New York. Therefore, the goals of this study conducted as a part of Integrated Marsh Management (IMM) project at Wertheim were to partially fill this knowledge gap and to assess the potential use of salt marsh aquatic insect fauna for biological monitoring. The Wertheim NWR EIMMVIM project sought to restore marsh hydrology and to preserve or enhance fish and wildlife habitat, while providing effective biological mosquito control. An additional goal was a reduction in the invasive reed, *Phragmites australis* (Cavanilles) Tinius ex Steudel (Poales: Poaceae), coverage (for more information on the project see Suffolk County 2009; Rochlin et al. 2009).

Aquatic insects were chosen as the focal community as their habitat was most directly affected by both marsh alterations and mosquito control measures such as pesticide applications. Additionally, this group was selected as a target assemblage due to ease of collection, readily available taxonomic keys and identification resources, existence of species at various trophic levels, and known bionomics for closely related freshwater taxa. The salt marsh aquatic habitat includes tidal creeks and ditches, mudflats (or pannes), large permanent ponds, and potholes (i.e. small pools). However, only the potholes, i.e. depressions with muddy bottom semi permanently filled with water, represent important habitat for truly aquatic insects on the salt marsh (Campbell and Denno 1978; Ward 1992). The potholes and pools also create the main larval habitat for salt marsh mosquitoes. This habitat is thus highly important from the mosquito surveillance and control perspective, and as such is most likely to be impacted by mosquito control measures. Therefore, it may be advantageous to use salt marsh pothole inhabitants as targets for biomonitoring. Monitoring pothole inhabitants could potentially be integrated into routine mosquito surveillance conducted by the majority of vector control districts along the Atlantic coast and may as a consequence, be exceptionally suitable for developing rapid bioassessment methods. Similar aquatic insect or macroinvertebrate based bioassessment protocols (e.g. index of biological integrity, IBI) have been extensively used in freshwater wetlands, especially those with standing water (EPA 2002b). An aquatic insect-based IBI adapted and adopted for tidal wetlands would provide a valuable tool for salt marsh health assessment.

## Materials and Methods

### Description of the study area

**Figure 1.**
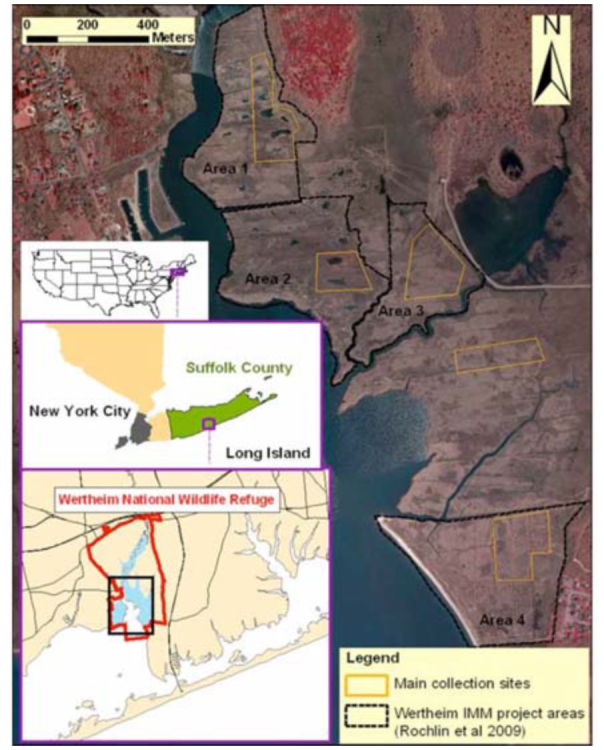
Collection sites for salt marsh aquatic invertebrates on Long Island, New York USA. High quality figures are available online.

**Figure 2.**
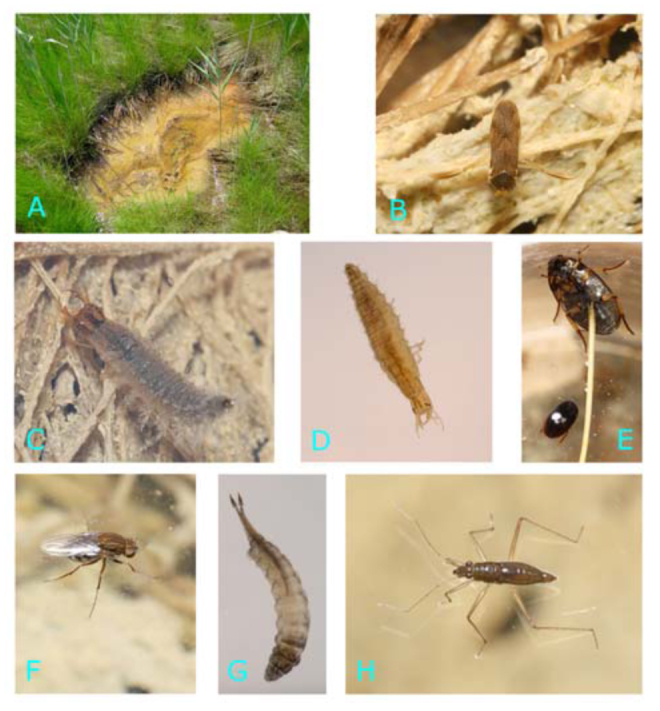
Common aquatic insects of the salt marsh pothole habitat. (A) a typical pothole in the *Spartina patens* high marsh. (B) *Trichocorixa verticalis*. (C) *Tropisternus quadristriatus* larva. (D) *Enochrus hamiltoni* larva. (E) *Tropisternus quadristriatus* adult (larger) and *Enochrus hamiltoni* adult (smaller). (F) Shore fly (Ephydridae) adult. (G) Shore fly (Ephydridae) larva. (H) *Mesovelia mulsanti.* High quality figures are available online

The study area was the Wertheim NWR IMM project sites, see Rochlin et al. (2009) for details. Briefly, the study collection sites included two salt marshes subjected to IMM changes including creating ponds, tidal creeks, and filling-in mosquito ditches performed 3–4 years prior to this study, and two unaltered control marshes with one additional unaltered site (Figure 1). Since a comprehensive faunistic survey (as opposed to comparisons among areas or impact assessment) was the main objective of this study, samples from all five areas were combined. Aquatic habitats ranging from relatively pristine (i.e. free of alterations and pesticide applications) to more impacted were thus included to provide more comprehensive picture of insect communities expected to be present elsewhere on the salt marshes subjected to different levels of environmental impact. Most landlocked potholes found in the *Spartina patens* (Aiton) Muhl (Poales: Poaceae) dominated high marsh (Figure 2), which were typically surveyed for immature stages of salt marsh mosquitoes within sampling locations (Rochlin et al. 2008), were also sampled for aquatic insects. Pothole salinities varied from of∼5∼15 ppt in the upper part of the high marsh and reached high values of ∼20 ppt in proximity to *Spartina alterniflora* Loiseleur, stands of the low marsh. The vegetation around potholes was dominated by *S. patens* with low form of *S. alterniflora*, *Distichlis spicata* (L.) Geeene, *Schoeno plectus* spp., and *P. australis* present at some locations.

Unaltered marshes within the study area (Area 3, Area 4, and an additional marsh between these two Areas, Figure 1) as well as one of the IMM marshes (Area 2, Figure 1) were treated with larvicides to control immature stages of salt marsh mosquitoes. Although fewer larvicide applications were made in the IMM marsh compared to the unaltered marshes (4 versus 5–6 per season), filling-in mosquito ditches in IMM Area 2 resulted in new mosquito larval habitat requiring additional control measures. Filling in mosquito ditches was a marsh surface restoration technique, which, together with the open marsh water management for mosquito control and tidal regime restoration for the invasive *P*. *australis* control, constituted the Wertheim NWR IMM [for more information and analysis see Rochlin et al. 2009]. Another IMM marsh (Area 1, Figure 1) did not support mosquito production post marsh alteration and was not treated in 2009.

Two biorational larvicides, defined as those with limited or no adverse effect on the nontarget organisms or the environment, were applied separately or in combination as needed. *Bacillus thuringiensis* var. *israelensis* (Bti; ValentBioscience Corporation, www.valentbiosciences.com) is a liquid bacterial product that kills early stages of mosquito larvae upon ingestion and is specific to mosquitoes in the concentrations applied to the salt marsh. Altosid Liquid Larvicide Concentrate (*S*-methoprene; Central Life Science, www.centrallifesciences.com) is an insect growth regulator that mimics juvenile hormones thus preventing mosquito pupae to molt into adult mosquitoes. Therefore, methoprene does not kill mosquito larvae immediately, allowing immature stages to remain in the food chain. Although methoprene appears to have broader nontarget impacts compared to Bti, its adverse effects on the insect salt marsh community are likely minimal and short lived (see Discussion).

### Sampling methods, processing, and identification

Approximately 125 to 175 total samples (i.e. 25 to 35 samples per collection site) were collected weekly from mid-May until midSeptember 2009, when the marsh surface was not flooded. All marshes were sampled prior to larvicide application during a given week. A standard mosquito dipper (350 ml) was used to collect aquatic insects from water surface (neustonic), water column (nektonic), and the bottom mud (benthic). A battery powered aspirator was occasionally used to collect adult Diptera from the water surface. The qualitative assessment of the insect abundance was performed in the field using the following categories: abundant widespread and abundant, common widespread but not numerous, rare - very localized distribution and/or few individuals found.

Samples were placed in plastic collection jars, brought to the laboratory, sorted, and identified. Some immature stages were kept alive in standard emergence jars to establish association between larval and adult stages. The remainder of the samples were preserved in 80% ethanol or pinned. Insects were identified to the family level using adult (Borror et al. 1989) or larval (Merritt and Cummins 1984) keys followed by generic and species determination for Collembola (Christiansen and Bellinger 1988), Heteroptera (Epler 2006), Coleoptera (Matta 1974; Michael and Matta 1977; Epler 1996), and generic identification for Diptera (McAlpine et al. 1981; McAlpine et al. 1987). Diptera species were identified using the following keys: Culicidae (Means 1979; Means 1987), Ceratopogonidae (Waugh and Wirth 1976), Dolichopodidae (Van Duzee 1923; Van Duzee 1926), and Ephydridae (Sturtevant and Wheeler 1954; Wirth 1964; Wirth 1971). Collembola and Ceratopogonidae specimens were mounted on slides for species identification. Species identification of Dolichopodidae and Ceratopogonidae were confirmed by the staff of the USDA Systematic Entomology Laboratory (Beltsville, MD). The presence of non-insect organisms (e.g. fish, worms, gastropods) in potholes was also qualitatively noted during sampling.

## Results

Weekly collections (n = 19, with very limited collection was possible in the last week in July and the first week of August due to complete marsh inundation.) resulted in over 2300 samples. Five hexapod orders and 11 families were collected over the course of the study. Diptera was the most diverse order with nine species from four families found during more than one week, followed by Coleoptera with four species from two families, Heteroptera with three species from three families, Colembola with one species, and Odonata with one species (Table 1). Excluding three mosquito species (i.e. *Culex salinarius* Coquillett, *Aedes cantator* Coquillett, *Aedes sollicitans* Walker (Diptera: Culicidae); see Rochlin et al. 2009 for distribution), only two out of the remaining 15 insect species were numerous in all collection sites throughout the study period. Water boatmen, *Trichocorixa verticalis* Fieber (Heteroptera: Corixidae), were the most abundant aquatic insect species with consistently high populations found in all study marshes (Figure 2). Adult shore flies, *Ephydra subopaca* Loew (Diptera: Ephydridae), were also commonly found on the surface of the potholes (Figure 2). The pupae and the pupal exuviae of this species were recovered by dipping near pothole edges, while the larvae inhabiting the floating algal mat (Kelts, 1979) were obtained only occasionally by this sampling method.

**Table 1.**
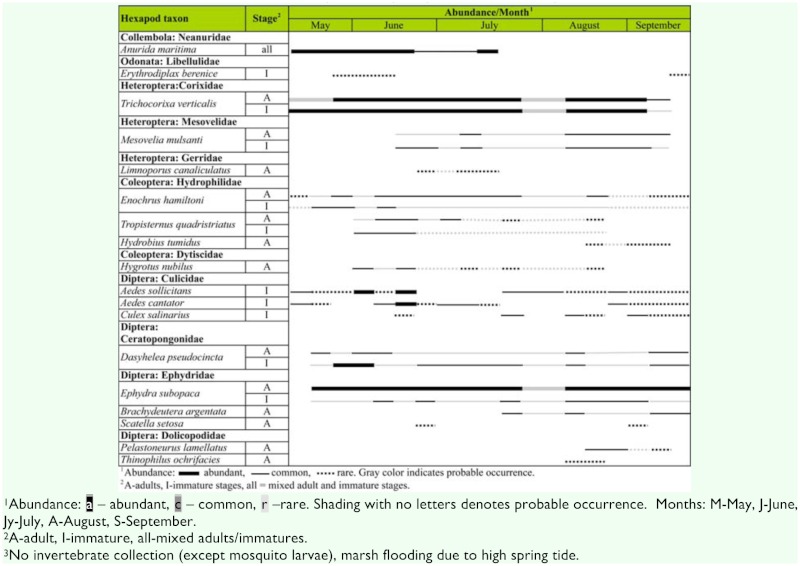
Seasonal distribution and abundance of aquatic hexapod species found on Long Island, New York, USA salt marshes.

A collembolan species, *Anurida maritima* Guérin-Méneville (Collembola: Neanuridae), was sometimes very abundant on the surface of some potholes, but this species' distribution in the pothole habitat was more localized and seasonally more restricted from May through early July. Another neustonic species, a water treader *Mesovelia mulsanti* White (Heteroptera: Mesovelidae), was common between mid-June and September and regularly found at vegetated edges of some potholes (Figure 2). Only the apterous form of adult *M. mulsanti* was collected during the study. A water scavenger beetle, *Enochrus hamiltoni* Horn (Coleoptera: Hydrophilidae), was consistently found in all study marshes (Figure 2). Adult beetles were collected more often than the larvae, whereas the opposite was true for a much larger hydrophilid species, *Tropisternus quadristriatus* Horn (Coleoptera: Hydrophilidae). The larvae of this species were commonly observed in June and early July, with the more cryptic adults collected infrequently through the second part of the summer (Figure 2). The pupae and the exuviae of a biting midge *Dasyhelea pseudocincta* Waugh & Wirth (Diptera: Ceratopogonidae) were sometimes very numerous in vegetated potholes. Although the attempts to emerge adults in the laboratory failed, the pupae were identified to the same species. Freshly emerged *D. pseudocincta* adults were collected on the water surface throughout most of the summer.

The remaining eight insect species were uncommon to rare. Naiads of the seaside dragonlet, *Erythrodiplax berenice* Drury (Odonata: Libellulidae), were only infrequently found in the pothole habitat over the study marshes. Since the adult *E. berenice* dragonflies were very common during July and August, it was likely that potholes did not constitute their main larval habitat in this salt marsh. A smaller shore fly species, *Brachydeutera argentata* Walker (Diptera: Ephydridae), was intermixed with *E*. *subopaca* in a few scattered locations where this species was relatively common. Another shore fly species, *Scatella setosa* Coquillett (Diptera: Ephydridae) - distinguished by small size and spotted wings, was rare. The dolicopodids, *Pelastoneurus lamellatus* Loew (Diptera: Dolichopodidae) and *Thinophilus ochrifacies* Van Duzee (Diptera: Dolichopodidae), were restricted to a small number of sites, with the former observed more frequently and in greater numbers. Similarly, the hydrophilid *Hydrobius* *tumidus* LeConte (Coleoptera: Hydrophilidae), the dytiscid *Hygrotus nubilus* LeConte (Coleoptera: Dytiscidae), and the water strider *Limnoporus canaliculars* Say (Heteroptera: Gerridae), had irregular spatial and limited seasonal distributions, although the dytiscid beetle, *H. nubilus*, was sometimes seen in higher numbers. The following taxa were rarely collected (mostly on a single occasion or as a single specimen): Entomobryidae and Sminthuridae spp. (Collembola), Saldidae sp. (Heteroptera), *Laccophilus maculosus* Say (Coleoptera: Dytiscidae) adult, *Hydrochara* spp. (Coleoptera: Hydrophilidae) larva, *Atrichopogon* spp. (Diptera: Ceratopogonidae) larva, Chloropidae spp. (Diptera) adult, *Eristalis* spp. (Diptera: Syrphidae) larvae, and Stratiomyidae spp. (Diptera) larvae.

Most of the insect species were found in the high marsh habitat with *Cx. salinarius* and *Ae*. *cantator* mosquitoes (Rochlin et al. 2008). This upper part of the salt marsh typically had salinities in 5–20 ppt range (mean ∼12.0 ppt) and *S*. *patens* as the dominant grass species. The brown salt marsh mosquito, *Ae*. *sollicitans,* also occurred abundantly at the upper high marsh, however, this salt tolerant species' habitat extended into the upper reaches of the low marsh with salinities similar to that of the bay (∼29 ppt). Very few aquatic insect species were found associated with *Ae. sollicitans* in this higher salinity habitat: *T. verticalis* and *E*. *subopaca* were commonly encountered, whereas *E*. *hamiltoni* and *B. argentata* were rarer.

Only two other invertebrate taxa, ribbon worms (Nemertea) and snails (Gastropoda), were commonly collected or observed in the salt marsh potholes, whereas amphipods and annelids were infrequent. A small insectivorous minnow, the mummichog (*Fundulus heteroclitus* L.), was commonly found in potholes throughout the study area. Shorebirds, such as lesser yellowlegs (*Tringa flavipes* Gmelin) and the least sandpiper (*Calidris minutilla* Vieillot) were also commonly observed foraging in the larger potholes.

## Discussion

### The salt marsh aquatic insect community

A total of 17 insect species and one hexapod species from 11 families and five orders were collected from the salt marsh potholes during this study (Table 1). Two dominant species, a corixid bug, *T*. *verticalis*, and a shore fly, *E*. *subopaca*, were abundant throughout the study period. Several dipteran species such as the salt marsh mosquitoes, *Ae. sollicitans* and *Ae*. *cantator* (Culicidae); a biting midge, *D*. *pseudocincta* (Ceratopogonidae), and a hydrophilid beetle, *E*. *hamiltoni* were commonly found in all study areas. Prior to the first prolonged drought in July, the salt marsh pothole assemblage was characterized by aquatic predators such as *E*. *berenice, H*. *nubilus, T quadristriatus*, and *E*. *hamiltoni* larvae as well as some neustonic species such as *A. maritima* and *L*. *canaliculatus*. The late season assemblage following the drought was characterized primarily by Diptera (ephydrids, dolichopodids, and culicids including *Cx*. *salinarius*) as well as increased abundance of *M*. *mulsanti*. Overall, the insect fauna composition of the Long Island, New York salt marsh pothole habitat was close to the salt marsh of New Jersey (Campbell and Denno 1978) despite much lower salinities observed in this study. It was also almost identical to the salt marsh aquatic insect community reported from New Hampshire (Kelts 1979).

The aquatic insect community of the Atlantic coastal marshes was characterized as depauperate by Campbell and Denno (1978) in their seminal study on the insect fauna of New Jersey salt marshes. Although 20 species of insects were found during their four-month survey, only seven were “regularly encountered”. Just one species (*T.verticalis*) was represented by more than 1000 individuals, three species were represented by ∼200 individuals each, and the remaining three species by ∼40 individuals each. Kelts (1979) found 15 aquatic insect species relatively common in New Hampshire salt marshes. Harsh environmental conditions (i.e. high salinity and temperature) and habitat instability (i.e. tidal action, wet/dry cycles, and wide fluctuations of physical and chemical parameters) were proposed as the main reasons for low diversity and species richness of the salt marsh aquatic insect communities (Foster and Treherne 1976; Campbell and Denno 1978; Kelts 1979; Robert and Matta 1984; Ward 1992).

High salinity (> 35 ppt) was proposed as a key factor limiting aquatic insects in salt marshes (Robert and Matta 1984). The authors found high salinity preceded the drying of the salt marsh and led to a statistically significant decline in invertebrate species richness. Higher salinity also appeared to have a negative effect on insect abundance. About five times as many insects emerged from a brackish portion of Maine salt marsh (mean salinity 9.5 ppt) than from a “true” salt marsh (mean salinity 30.1 ppt), with both experiencing similar average temperatures and fluctuations (MacKenzie 2005). These observations may explain the discrepancy between the Campbell and Denno (1978) study, which found very impoverished salt marsh insect fauna, and others that found more commonly occurring insect species (Wall 1973; Kelts 1979; Robert and Matta 1984; MacKenzie 2005; this study). While Campbell and Denno (1978) reported an average salinity of about 37.0 ppt, most other surveys were conducted in lower salinities in the 10–30 ppt range. In the present study, most aquatic insects were recovered from areas with moderate salinities between 7.0 and 17.0 ppt (with mean of ∼12.0 ppt) closely associated with the larval habitat of *Cx*. *salinarius* and *Ae. cantator* mosquitoes (Suffolk County 2009; Rochlin et al. 2009). Very few aquatic insects with the exception of *T*. *verticalis*, *E. hamiltoni*, and the ephydrid flies were collected in pools with notably higher salinity (∼14 ppt to more than 30 ppt). This high salinity pothole habitat was associated with another abundant salt marsh mosquito species, *Ae. sollicitans* (Means 1979; Suffolk County 2009).

Another environmental factor, a tidal regime (i.e. alternating wet and dry periods) was also proposed as the main reason responsible for low aquatic insect diversity in a salt marsh (Robert and Matta 1984). The authors noted that the species diversity decreased before and during droughts and quickly rebounded following salt marsh re-flooding. Seasonal drying of the marsh during the second part of the summer was thus responsible for the disappearance of many invertebrate species that colonized the marsh early in the season. A similar trend was observed in this study with fewer species and/or individuals found in the late summer and early fall after the summer droughts. Alternatively, this late summer decline in aquatic insect species diversity was attributed to increased water temperature and decreased level of oxygen by some researchers (Campbell and Denno 1978).

**Figure 3.**
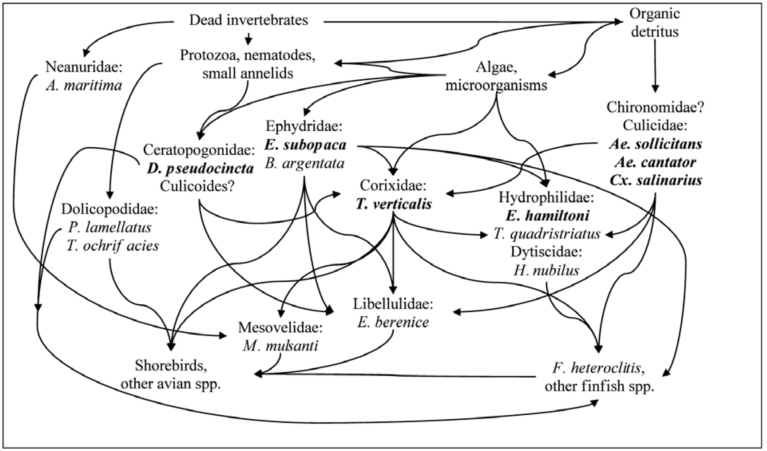
Schematic diagram of the major food chain relationships for aquatic insects in salt marsh potholes. Two top vertebrate predator groups (i.e. killifish and birds) are also shown (modified from Kelts 1979; also Hungerford 1917; Joosse 1976; Liney 1976; Campbell 1979; Merritt and Cummins 1984; LaSalle and Bishop 1990; and personal observations). Commonly encountered and abundant species are indicated in bold letters. Question mark denotes groups requiring additional survey. High quality figures are available online

Despite some dissimilarities between aquatic insect communities along the longitudinal geographic transect from Maine to Virginia, the species composition remained surprisingly comparable among several studies (Wall 1973; Campbell and Denno 1976; Campbell and Denno 1978; Kelts 1979; Robert and Matta 1984; MacKenzie 2005). Based on these similarities, the proposed aquatic insect community structure in the Long Island, New York salt marshes was schematically depicted within the major food web relationships (Figure 3). Similar to the New Jersey and New Hampshire findings (Campbell and Denno 1976; Campbell and Denno 1978; Campbell 1979; Kelts 1979), the Long Island, New York salt marsh pool insect fauna was typically dominated by the omnivorous corixid, *T*. *verticalis* that was commonly encountered in all collection sites from late spring through early fall. Due to its abundance, *T*. *verticalis* was centrally important in the aquatic food chain (Kelts 1979). This species could tolerate a wide range of salinity and temperature (Kelts 1979) and was also less affected by fish predation — the principal biotic factor that shapes salt marsh invertebrate community, apparently due to the unpalatability of the late instars and adults (Campbell and Denno 1978; Kelts 1979). While the early instars are mostly herbivorous, late instars and adults are predacious with chironomid midges as the main prey item (Campbell 1979; Kelts 1979). Other relatively common carnivorous species included nektonic hydrophilid *T*. *quadristriatus* larvae, libellulid *E*. *berenice* naiads, and neustonic mesovelid *M*. *mulsanti.* The heteropteran, coleopteran, and odonatan predators consumed mainly various immature dipterans that were common to abundant in the salt marsh pothole habitat. In turn, the Diptera species occupied several lower ecological niches in the food chain, from detritivores (Culicidae, Chironomidae) to herbivores (Ephydridae, *Dasyhelea* spp.) and predators on small aquatic organisms (Dolichopodidae, some Ceratopogonidae). Fish were the most important top insect predators in the pothole communities (Campbell and Denno 1978; personal observations), and both invertebrate and vertebrate prey were also consumed by shorebirds (Kelts 1979; personal observations).

Although salt marsh chironomids were reported by a number of studies (Wall 1973; Campbell and Denno 1978; Kelts 1979; Robert and Matta 1984; MacKenzie 2005), no immature Chironomidae were recovered during this study. The spatial distribution of salt marsh chironomid larvae appeared to be very patchy (Campbell and Denno 1978) and they were best collected by specialized benthic sampling equipment or emergence traps (MacKenzie 2005). The adults were only rarely encountered on the salt marsh vegetation (Kelts 1979; this study). These results underscored the main limitation of this study, namely the collection technique. Dipping was not very effective in obtaining benthic organisms, and while adequate for a rapid qualitative survey, this technique might not be suitable for quantitative studies. More quantitative but also more labor intensive methods for sampling salt marsh insects were described previously (Campbell and Denno 1978; Kelts 1979). These techniques may be adapted for comparative and impact assessment studies of the salt marsh insect fauna.

### Potential utility of salt marsh aquatic insects as bioindicators

Ecologic (or biological) indicators are used to detect environmental change due to anthropogenic or natural causes and to assess the condition of a coastal ecosystem (Niemi et al. 2004). The main premise of using bioindicators is that the animal community reflects the health of a wetland, and responds to disturbances by measurable changes in biological attributes such as diversity, taxonomic richness, trophic structure, or impact on individual organisms (EPA 2002a). Freshwater aquatic macroinvertebrates, and especially insects, have long been among the most utilized taxonomic assemblages for biological assessments due to abundance, localized life cycle, important trophic position, and known responses to many environmental stressors (EPA 2002b). A number of insect-based indices of biological integrity (IBI) such as Ephemeroptera, Plecoptera, and Trichoptera (EPT) index were developed for freshwater wetlands. However, the use of aquatic insects for assessing salt marsh health has been sporadic and lacks a systematic approach, with no standardized insect— or invertebrate—based IBI currently in existence for tidal wetlands. This is unfortunate given the potential usefulness of salt marsh aquatic insects for monitoring and evaluating two principal environmental impacts common in many developed coastal areas such as Long Island, New York, i.e. anthropogenic disturbance and pesticide application for mosquito control. Anthropogenic disturbance may include both negative (e.g. marsh degradation, runoff) as well as positive (e.g. marsh restoration) changes from the natural resource management standpoint, which may directly affect the marsh community and its trophic structure. Taxonomic richness of salt marsh aquatic insects and the response by individual indicator species may be used for assessing environmental effects (Table 2). Aquatic insects may prove even more useful for monitoring impact of mosquito larvicides due to close taxonomic relationship, similar physiologies, and comparable body size of many insect species. A good indicator species should display sufficient abundance, ease of collection, suitable late summer—early fall seasonality coinciding with the peak pesticide usage, and either direct or indirect response to the pesticides (Tables land 2). Aquatic insects appeared to be the only affected taxonomic group among both invertebrates and vertebrates by Bti and methoprene (the two most common salt marsh larvicides) in the only long—term, and one of the most comprehensive ecological studies on the effects of these larvicides in a wetland community (Hershey et al. 1998; Niemi et al. 1999; Niemi et al. 2004). The follow-up study revealed statistically detectable negative effects limited to some nematoceran Diptera such as chironomid midges, but not in any other insect group (Balcer et al. 1999).

**Table 2.**
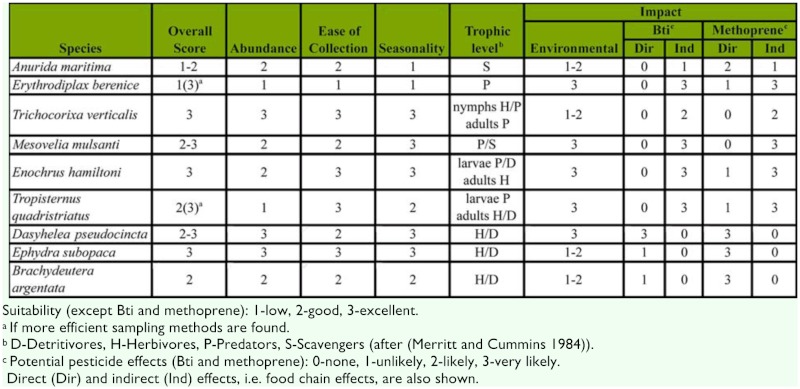
Evaluation of the utility of common aquatic hexapod species occurring on Long Island (NY) salt marshes for biomonitoring.

A promising group for salt marsh aquatic insect IBI are the hydrophilid beetles, *E*. *hamiltoni* and *T*. *quadristriatus*, which are well suited for both pesticide and environmental impacts monitoring. Although likely not affected by Bti (Hershey et al. 1998), some studies found reductions in either larval (Hershey et al. 1998) or adult (Steelman et al. 1975) hydrophilid populations following methoprene treatment. These results are equivocal, since other studies failed to detect any visible methoprene effects in the field (Miura and Takahash 1973; Balcer et al. 1999). However, as one of the main invertebrate predators, hydrophilid larvae can be expected to respond to significant reductions in their prey such as chironomid midges and mosquitoes. In addition, together with other important predatory species such as *M*. *mulsanti* and *E*. *berenice*, *E*. *hamiltoni* and *T*. *quadristriatus* may be more sensitive to environmental changes due to more restrictive habitat requirements and higher trophic position in the aquatic food chain (Figure 3).

The two most common aquatic salt marsh insects, the corixids (*T*. *verticalis*) and the ephydrid flies (*E. subopaca*), are probably less useful for environmental impact monitoring due to their high tolerance and broad habitat requirements. These two species scored high on sufficient abundance, ease of collection, and suitable late summer-early fall seasonality (Table 1). Although both Bti and methoprene appeared to have no direct effect on corixids (Miura and Takahash 1973; Breaud et al. 1977; Garcia et al. 1980; Lawler et al. 2000), the abundance of the late-instar and adult water boatmen in the salt marsh potholes was directly correlated with the abundance of their main prey, the larvae of chironomid midges (Campbell 1979). Bti and methoprene are likely to have the most evident non-target impact on Diptera in general (Hershey et al. 1998), and especially on Chironomidae as demonstrated by several studies (Miura and Takahash 1973; Breaud et al. 1977; Hershey et al. 1998; Balcer et al. 1999). The most easily detectable salt marsh dipteran species, the ephydrid flies (*B. argentata* in this case), exhibited significantly elevated mortality when exposed to methoprene in laboratory (Miura and Takahash 1973), but limited outdoor experiments failed to demonstrate clear methoprene or Bti effects (Miura and Takahash 1973; Garcia et al. 1980). Despite these inconclusive results underscoring the need for future research, Ephydridae (especially *E*. *subopaca* and *B*. *argentata)* remains a strong candidate as an indicator taxon for potential direct Bti/methoprene effects.

Another very promising Diptera taxon is biting midges, Ceratopogonidae, specifically the genus *Dasyhelea*. Despite being classified as “a biting midge”, *Dasyhelea* adults do not bite and are relatively common in salt marshes, with a number of species originally described from Long Island, New York (Waugh and Wirth 1976). As nematoceran Diptera, ceratopogonids are expected to be toxicologically susceptible to both Bti and methoprene. Indeed, ceratopogonids experienced high mortality under Bti treatment in outdoor exposures (Garcia et al. 1980). Ceratopogonid abundance was negatively affected by both Bti and methoprene treatments in freshwater wetlands (Hershey et al. 1998); however, the follow-up study failed to detect any pesticide effects (Balcer et al. 1999).

The dragonfly naiads (*E*. *berenice*) are not likely to be directly affected by either Bti or methoprene (Miura and Takahash 1973; Breaud et al. 1977; Garcia et al. 1980; Purcell 1981; Aly and Mulla 1987). Indirect negative impacts of methoprene on libellulid dragonfly naiads were previously observed (Steelman et al. 1975; Breaud et al. 1977); however, this effect was not always detectable (Norland and Mulla 1975). As the top aquatic invertebrate predator in salt marshes (Kelts 1979; Figure 3), *E*. *berenice* would have been an excellent candidate as a bioindicator. Unfortunately, low numbers of naiads were recovered in this and most other studies (Campbell and Denno 1978; Robert and Matta 1984) necessitating significantly improved collection techniques to fully utilize this species for biological monitoring. Contrary to the more cryptic *E*. *berenice*, the water treader *M*. *mulsanti* can be collected in sufficient, albeit moderate, numbers.

Thus, the list of the potential indicator species at a higher trophic level (Figure 3) includes representatives from each benthic (*E*. *berenice*), nektonic (*E. hamiltoni*, *T. quadris triatus*), and neustonic (*M*. *mulsanti*) microhabitats in salt marsh potholes. Lower trophic levels are represented by more omnivorous *T. verticalis*, nektonic/benthic herbivores, or detritivores: *E*. *subopaca*, *B*. *argentata*, and *D*. *pseudocincta*, and a neustonic collembolan *A*. *maritima*. The latter may also be sensitive to methoprene similarly to other Collembola species (Campiche et al. 2007) and may be indirectly affected through the food chain as a generalist invertebrate scavenger by both Bti and methoprene.

Missing from this list is the most numerous nematoceran Diptera family, Chironomidae, which is expected to be susceptible to both Bti and methoprene due to its very close taxonomic relationship with mosquitoes (Hershey et al. 1998). In fact, products containing either Bti (e.g. VectoBac 12AS, Valent Biosciences) or methoprene (e.g. Strike, Wellmark International, www.wellmarkinternational.com) are registered for use against nuisance chironomid midges. Not surprisingly, there is strong evidence that Chironomidae can be impacted by Bti and methoprene under field conditions (Miura and Takahash 1973; Breaud et al. 1977; Hershey et al. 1998; Balcer et al. 1999). However, this negative effect appears to be limited to certain tribes or species within this dipteran family based on the results of the two published longterm ecological studies (Balcer et al. 1999; Lundstrom et al. 2009). Therefore, using chironomids for biomonitoring may require species identification, which may be difficult without properly trained taxonomists involved and would certainly necessitate extensive benthic sampling employing specialized equipment and techniques. In addition, any major impact on Chironomidae is expected to have a serious effect on other species, especially those at higher trophic levels, and thus should be detectable. Elucidating species richness of salt marsh Chironomidae will certainly require a separate and more extensive investigation.

The present study is only the initial step in assessing Long Island, New York salt marsh invertebrate diversity and developing invertebrate—based metrics for salt marsh health monitoring. More taxonomic work is needed to include invertebrates other than hexapods, and more ecological and toxicological research is required to understand how individual species respond to different environmental stressors. Determining quantitative sampling procedures and selecting most responsive biological or ecological attributes to use as metrics will be necessary to complete salt marsh invertebrate IBI (EPA 2002b). Although laborious and complex, the outcome holds a great potential to generate a common salt marsh invertebrate index applicable to a wide geographic range of tidal wetlands on the Atlantic coast of North America.

## **Abbreviations**

Bti: *Bacillus thuringiensis* var. *israelensis*
IBI: index of biological integrity
IMM,: integrated marsh management
